# Perturbation-Induced Stepping Post-stroke: A Pilot Study Demonstrating Altered Strategies of Both Legs

**DOI:** 10.3389/fneur.2019.00711

**Published:** 2019-07-03

**Authors:** Katherine M. Martinez, Mark W. Rogers, Mary T. Blackinton, M. Samuel Cheng, Marie-Laure Mille

**Affiliations:** ^1^Department of Physical Therapy and Human Movement Sciences, Northwestern University Feinberg School of Medicine, Chicago, IL, United States; ^2^Department of Physical Therapy and Rehabilitation Science, University of Maryland School of Medicine, Baltimore, MD, United States; ^3^Physical Therapy Program, Nova Southeastern University, Tampa, FL, United States; ^4^Physical Therapy Program, Nova Southeastern University, Fort-Lauderdale, FL, United States; ^5^UFR-STAPS, Université de Toulon, La Garde, France; ^6^Institut des Sciences du Mouvement (ISM UMR 7287), Aix Marseille Université and CNRS, Marseille, France

**Keywords:** postural control, reactive balance, compensatory stepping, rehabilitation, stroke

## Abstract

**Introduction:** Asymmetrical sensorimotor function after stroke creates unique challenges for bipedal tasks such as walking or perturbation-induced reactive stepping. Preference for initiating steps with the less-involved (preferred) leg after a perturbation has been reported with limited information on the stepping response of the more-involved (non-preferred) leg. Understanding the capacity of both legs to respond to a perturbation would enhance the design of future treatment approaches. This pilot study investigated the difference in perturbation-induced stepping between legs in stroke participant and non-impaired controls. We hypothesized that stepping performance will be different between groups as well as between legs for post-stroke participants.

**Methods:** Thirty-six participants (20 persons post-stroke, 16 age matched controls) were given an anterior perturbation from three stance positions: symmetrical (SS), preferred asymmetrical (PAS−70% body weight on the preferred leg), and non-preferred asymmetrical (N-PAS−70% body weight on the non-preferred leg). Kinematic and kinetic data were collected to measure anticipatory postural adjustment (APA), characteristics of the first step (onset, length, height, duration), number of steps, and velocity of the body at heel strike. Group differences were tested using the Mann-Whitney *U*-test and differences between legs tested using the Wilcoxon signed-rank test with an alpha level of 0.05.

**Results:** Stepping with the more-involved leg increased from 11.5% of trials in SS and N-PAS up to 46% in PAS stance position for participants post-stroke. Post-stroke participants had an earlier APA and always took more steps than controls to regain balance. However, differences between post-stroke and control participants were mainly found when stance position was modified. Compare to controls, steps with the preferred leg (N-PAS) were earlier and shorter (in time and length), whereas steps with the non-preferred leg (PAS) were also shorter but took longer. For post-stroke participants, step duration was longer and utilized more steps when stepping with the more-involved leg compared to the less-involved leg.

**Conclusions:** Stepping with the more-involved leg can be facilitated by unweighting the leg. The differences between groups, and legs in post-stroke participants illustrate the simultaneous bipedal role (support and stepping) both legs have in reactive stepping and should be considered for reactive balance training.

## Introduction

Sensorimotor dysfunction is a common outcome after a stroke that contributes to deficits in gait and voluntary stepping as well as impaired postural control (1–6). Stroke survivors initiate gait predominantly with their more-involved leg and display asymmetrical cadence and step kinematics (7–9). When balance is challenged, stepping is a common strategy used to maintain upright control (10, 11). Previous studies have shown that older adults rely more on stepping to recover their balance and this induced stepping strategy is less effective for those who have diminished sensorimotor function in their lower limbs (11–14). Dynamic balance is also impaired in stroke survivors compared to controls as seen in number of falls, greater sway and altered ground reaction forces after a lateral perturbation and differences during automatic and voluntary weight shifts (15–17). Whether stepping voluntarily as in gait or reactively in response to a perturbation, the body must coordinate the use of both lower limbs, one for support and one for stepping. When presented with an asymmetrical impairment of the lower limb after stroke, the question becomes which leg should be more effectively and safely used for support vs. stepping and whether it possible to induce the use of a specific leg?

Several studies on perturbation stepping in persons post-stroke report a predilection for initiating steps with the less-involved leg, even with lateral perturbations (18–22). Two studies involving steps with the paretic (more-involved) leg with cuing found no difference in step onset or number of steps between paretic and non-paretic (less-involved) legs during these cued reactive stepping tasks (23, 24). Our previous study using a forward-diagonal perturbation method to induce stepping with paretic leg in stroke survivors found slower and earlier steps for the paretic leg and that induced steps were faster and earlier compared to voluntary stepping (20). However, studies on reactive stepping report no difference in the incidence of falls when initiating a step with paretic or non-paretic leg (19, 21, 23).

The asymmetry in stepping preference and sensorimotor function creates challenges in investigating the bipedal nature of induced reactive stepping. Comparing perturbation induced reactive stepping responses between individuals' post-stroke and controls would provide some insight into the alterations in inter-limb control of reactive stepping. To our knowledge, only one study has compared persons post-stroke and controls and only during a posterior perturbation. In that study, post-stroke participants initiated fewer backward steps, took more steps when they did initiate stepping, and were less stable with shorter step length and delayed onset compared to controls (25). However, the extent to which these changes in stepping performance affect balance recovery after stroke for other directions of stepping remains to be determined.

Differences between the inter-limb control of stepping for different directions of imbalance in unimpaired controls and persons post-stroke are important to identify given the bipedal nature of the induced stepping task and the direction-dependent neuromotor requirements for recovering balance (13). Incomplete understanding of the inter-limb step characteristics during induced stepping after stroke limits the design of effective treatment interventions to improve balance function and reactive postural control. For example, additional information about the use of the more-involved and less-involved legs prior to and during stepping as well as after step landing will help delineate the effectiveness of each leg in the different phases of balance recovery.

To further address the foregoing issues, the purpose of this pilot study was to investigate potential differences in reactive perturbation-induced stepping characteristics between: (1) post-stroke survivors and healthy control participants, and (2) between legs in the post-stroke participants. Using a simple method of modifying the initial stance symmetry, we hypothesized that for perturbation induced reactive steps in the anterior direction: (1) stance asymmetry will alter the selection of the stepping leg, (2) stepping performance would be different between post-stroke participants and age-matched controls for both legs, and (3) the more-involved leg would be less efficient in reactive step performance than the less-involved leg.

## Methods

Data sharing is fully appreciated. The raw data supporting the conclusions of this manuscript will be made available by the corresponding author, without undue reservation, to any qualified researcher upon reasonable request.

### Study Population

Thirty-six individuals (20 persons post-stroke and 16 controls) participated in this study. The post-stroke participants were recruited from the Clinical Neuroscience Research Registry. They all met the following criteria: history of a single unilateral non-cerebellar stroke at least 1 year prior to the study, ambulated independently in the community and did not use a wheelchair or a long leg brace. Additional selection criteria included: no history of other neurological and orthopedic diseases or surgery to the lower extremities, and not currently receiving occupational or physical therapy. In addition, post-stroke participants were physically screened for the ability to stand and walk independently 10 feet without an assistive device or orthosis, unilateral paresis, ability to give consent. They were excluded if they scored below a five on the Functional Ambulation Categories (26, 27). Controls were recruited from the Northwestern University Buehler Center Aging Research Registry and from flyers posted on campus. They were age (± 3years) matched without neurological or orthopedic impairments. All participants gave written inform consent for the study which was approved by the Institutional Review Board of Northwestern University and Nova Southeastern University and performed in agreement with the ethical principles of the Declaration of Helsinki. Participant demographics characteristics are presented in Table 1.

**Table 1:** Participants characteristics.

**Demographics**							**Clinical assessments**							
**Participants**	**Gender**	**Paresis**	**Age** **(years)**	**Height** **(cm)**	**Weight** **(Kg)**	**Time since stroke** **(years)**	**ABC** **(out of 100%)**	**TUG** **(s)**	**Step test** **(n)**		**Sensations** **(g)**		**UMC**	
**Post-stroke (*****n*** **=** **20)**									***Non-PSL***	***PSL***	***Non-PSL***	***PSL***	***Non-PSL***	***PSL***
PS01	Male	Right	42	175	80	7.2	87.5	8.5	13	13	10	2	1	1
PS02	Male	Right	64	177	68	11.0	86.9	11.2	8	14	10	2	1	1
PS03	Male	Left	44	180	61	10.0	85	16	16	13	0.4	0.4	2	2
PS04	Male	Right	64	184	85	5.6	88.8	13.1	9	14	2	0.4	1	1
PS05	Male	Right	69	171	81	19.5	73.1	17.6	6	7	2	0.4	1	2
PS06	Male	Right	59	178	88	10.3	90	10.2	7	13	2	2	1	1
PS07	Male	Left	58	186	76	18.5	78.1	9	11	13	4	2	1	1
PS08	Female	Left	37	164	77	6.6	69.4	12.3	6	7	0.4	10	1	1
PS09	Female	Right	65	162	60	11.3	55	16.9	4	4	10	2	3	1
PS10	Male	Right	50	167	68	13.0	95	8.6	10	14	0.4	0.4	1	1
PS11	Female	Right	21	170	54	1.4	94.4	8.8	9	9	2	2	2	1
PS12	Male	Right	49	182	98	26.8	70	14.8	9	13	2	2	2	1
PS13	Male	Left	49	167	89	16.4	85	16.5	7	8	2	2	3	2
PS14	Male	Left	60	179	98	3.6	95	16.3	5	8	2	0.4	1	1
PS15	Female	Right	73	168	86	9.5	81.8	14	9	10	0.4	2	1	1
PS16	Male	Right	64	183	100	6.3	78.8	15.1	7	8	4	4	3	1
PS17	Male	Right	56	174	79	4.3	80.6	11.6	12	12	2	2	1	1
PS18	Female	Right	49	165	55	3.9	82.2	10.4	8	10	2	2	2	1
PS19	Male	Left	65	172	96	2.0	90.6	23.5	6	5	4	4	3	1
PS20	Male	Right	56	178	96	3.1	76.3	17.1	7	8	4	0.4	1	1
Mean			54.7	174.1	79.8	9.5	82.2	13.6	8.45^#^	10.1^#^	3.3	2.1	1.6^#^	1.2^#^
(*SD*)			(12.4)	(7.2)	(14.8)	(6.7)	(10.1)	(3.9)	(2.9)	(3.2)	(3.1)	(2.1)	(0.82)	(0.37)
Median			57	174.5	80.5	8.4	83.6	13.6	8	10	2	2	1	1
[range]			[21-73]	[162 186]	[54 100]	[1.4 26.8]	[55 95]	[8.5 23.5]	[4 16]	[4 14]	[0.4 10]	[0.4 10]	[1 3]	[1 2]
**Controls (*****n*** **=** **16)**														
Mean			52.8	172.6	75.7		92.8^*^	8.3^*^	17.1^*^	17.6^*^	1.2^*^	1.6	1^*^	1
(*SD*)			(16.4)	(7.4)	(16.2)		(11.05)	(1.3)	(3.3)	(3.1)	(1.1)	(1.2)	(0)	(0)
Median			56	171	72		96.4	8.1	16.5	17	2	2	1	1
[range]			[22 76]	[160 189]	[54 110]		[57.5 100]	[6.1 10.6]	[13 23]	[14 22]	[0.4 4]	[0.4 4]	[1]	[1]

*ABC, Activity-specific Balance Confidence Scale; TUG, Timed Up and Go test; PSL, preferred stance leg = less-involved leg (without paresis) for participants post-stroke; UMC, Upright Motor Control test*;

* *indicates significant difference between groups (Man-Whitney U-test)*,

# *indicates significant difference between more-involved (non-PSL or paresis side) and less-involved legs (PSL) in stroke (p < 0.05) (Wilcoxon signed-rank test)*.

### Protocol

#### Clinical Assessments

The clinical tests were selected to give a fuller picture of the participants' characteristics (Table 1). The Activity-specific Balance Confidence scale (ABC) questionnaire was completed to assess balance confidence in performing 16 functional activities using a rating of 0% (no confidence at all) to 100% (complete confidence) (28–30). Scores above 80 indicated high balance confidence. The Timed Up and Go test (TUG) was used to assess balance and ambulation mobility (31, 32). Participants began seated and walk three meters at their normal pace, turn around, walk back, and sat down. The Step Test (ST) assesses stance stability and dynamic balance in persons post-stroke (33, 34). The participants started in standing and place their whole foot up on a step 7.5 centimeters high as many times as they could in 15 s without loss of balance, first with their more-involved leg and then with their less-involved leg. The score for each leg was the number of times the participant touches the step in 15 s.

Sensorimotor measures included sensation on the plantar surface of feel and extension control in standing. Deep pressure sensation in the feet were measured using Semmes-Weinstein aesthesiometer (35, 36). The aesthesiometer filaments were placed perpendicular to the plantar surface of the first ray. The lowest gram filament perceived when touched to the foot was recorded. Testing strength in persons post-stroke is complicated due to the presence of synergy patterns, altered motor recruitment patterns, and spasticity in some patients. The Upright Motor Control (UMC) extension test provides information on the ability to weight bear and extend the leg in person post-stroke (37–39). In this test the participant bend both knees approximately 30° with light upper extremity support for balance and then lifts their leg off the ground. In this single leg support position, the ability of the involved knee to extend is graded strong (scored as 1) if able to fully extend, moderate (scored as 2) if able to support on flexed knee, and weak (scored as 3) if unable to support on flexed knee. The closed chain position is similar to the leg extension position needed for the landing phase of the induced step.

#### Perturbation-Induced Stepping

**Figure 1:**
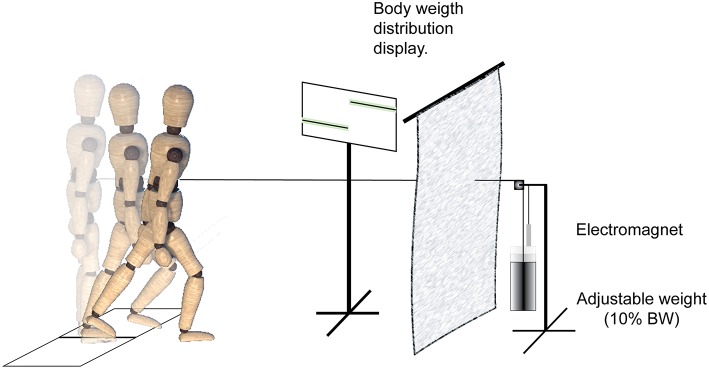
Experimental setup and protocol. A mechanical weight-drop waist-pull perturbation was applied in the anterior direction. When the participants maintained a pre-instructed weight-bearing load on the force plate for 250–1,000 ms, it released an electromagnet and triggered the free-fall of a weight equal to 10% of the subject body weight. The perturbation device was behind a screen, and a monitor provided visual feedback to the subjects on their standing weight-bearing symmetry.

Participants stood with feet shoulder-width apart, each foot on a separate force platform (Figure 1). They were placed in a safety harness attached by straps to an overhead rigid beam that minimized potential falls but allowed them to move freely. The placement of the feet was traced to allow the subject to return to the same foot position for each trial. A belt linked to a cable connected to a perturbation device was secured to the subject's waist, and the cable height was adjusted to the level of the umbilicus. Stepping was induced using a mechanical weight drop system hidden behind a screen that delivered an anterior perturbation equal to 10% of the individual's body weight (BW). A monitor provided visual feedback to the subjects on their body weight distribution and the pull was triggered when the subject maintained the required weight distribution (see below) on the force plate for 250–1,000 ms. Participants were instructed to react naturally and not resist the waist pull perturbation.

**Figure 2:**
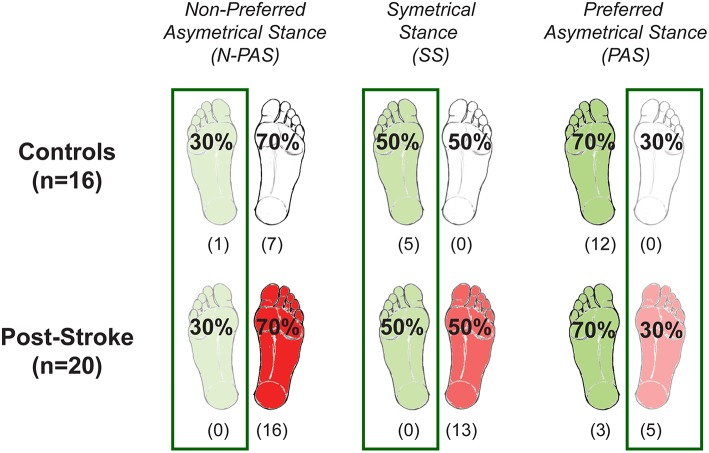
Body weight distribution conditions. The three different weight-bearing (WB) conditions are represented here for the control and post-stroke participants. The preferred supporting leg is presented in green and the more-involved leg is presented in red for post-stroke paticipants. The numbers between parentheses indicates the number of participants who never stepped with that leg for any of the trials of that condition. Based on these numbers, the difference between groups was tested only when stepping with the preferred/less-involved leg for the N-PAS and SS conditions, and with the non-preferred/more-involved leg for the PAS condition, whereas difference between legs was tested in the N-PAS and SS conditions for controls, and for stroke participants in the condition with the same body weight distribution (i.e., less-involved in the N-PAS condition and more-involved in the PAS condition).

Three different body weight distributions were used to encourage stepping with both legs (Figure 2). In the symmetrical stance (SS) condition, participants placed 50% (±3%) of their weight on each foot. In the preferred asymmetrical stance (PAS) condition, they placed 70% (±3%) of BW on their preferred supporting leg (i.e., the one that controls naturally selected for single limb stance and the less-involved limb for the post-stroke participants). In the non-preferred asymmetrical stance (N-PAS) condition, they were asked to put 70% (±3%) of BW on the non-preferred supporting leg (i.e., the more-involved limb for the post-stroke participants). These values were based on the abilities of post-stroke participants to shift weight onto their involved leg in prior studies and in pilot testing (40, 41).

After three practice trials of increasing weight bearing to the predetermined level, 10 trials at each of the three weight distribution conditions and five catch trials (i.e., perturbation at only 2% BW) were presented in the same predetermined standard randomized order. Participants were given a seated rest after 18 trials or as needed. A research personnel was always standing next to the participants for safety.

### Data Recording

Two force platforms (AMTI, OR6-6, Newton MA, USA) recorded the forces under each foot and allowed the calculation of the total center of pressure (CoP) position. Reflective markers were attached bilaterally over key landmarks of the leg and trunk (on the medial and lateral malleolus, calcaneus, lateral first and fifth metatarsal head, top of second metatarsal head, medial and lateral femoral condyles, and bilateral acromion) to record foot, shank, and trunk movements using an eight-camera motion analysis system (QTM-Qualisys Tracking Manager, Qualisys, Gothenburg, Sweden). Perturbation, kinetic, and kinematic data was collected in Qualisys at 100 Hz for 15 s, beginning at least 1 s prior to perturbation. Kinematic data was filtered using a second order Butterworth filter with 1 bidirectional pass resulting in a fourth order filer. The cutoff frequency was set at 10 Hz (42, 43).

### Dependent Variables

Following the perturbation, pre-step postural activity, referred to as anticipatory postural adjustment (APA), is often characterized by an initial displacement of the CoP toward the stepping leg that initiates weight transfer toward the upcoming single stance leg (12). Its characteristics were derived from the net mediolateral CoP displacement. The APA onset was defined as the beginning of the CoP displacement (i.e., when the first derivative becomes continuously >0). The APA amplitude was the maximum displacement of the net CoP toward the stepping side, and the APA duration was from the onset of the CoP displacement to the APA Peak.

For each trial, the execution of the step was evaluated by determining the number of steps, first step onset timing, first step length, width, height, and duration. These characteristics were identified from the ankle markers of the stepping side. The beginning and end of the step were identified from the vertical velocity of the marker in order to determine the step duration and the mediolateral and anteroposterior displacement of the foot (i.e., step width and length). The step clearance was defined as the maximal vertical excursion of the step ankle marker above the ground. The onset time of stepping was calculated relative to the onset of the perturbation.

To quantify the termination of the whole-body movement which influences the maintenance of balance and the necessity of a second step, the velocity of the body at the end of the first step (first heel strike) was calculated using the midpoint of the bilateral acromion markers. The velocity was multiplied by the subjects' mass to calculate the body momentum as the difference in momentum between first and second heel strike.

### Statistics

Descriptive statistics were used to describe the data for both groups and are reported as median (Mdn) and interquartile range (25 and 75th percentile). The Shapiro-Wilk and Levene tests indicated that the reactive step measures were not normally distributed, and that the variance was different between groups. In addition, an unequal number of stepping responses between groups and legs were observed (Figure 2), thus requiring non-parametric analyses. Non-parametric tests were also used for the clinical measures.

Based on the number of participants who took steps with each leg (Figure 2), the difference between groups was tested separately for each stepping leg using the Mann-Whitney U-test. We compared the groups when stepping with the preferred/less-involved leg for the N-PAS and SS conditions, and with the non-preferred/more-involved leg for the PAS condition. Differences between legs were assessed using a Wilcoxon signed-rank test in SS conditions in controls, whereas for post-stroke participants, we compared stepping performance between the legs for the same body weight distribution condition. Thus, steps with the less-involved leg in the N-PAS condition (i.e., less weight on the stepping leg) were compared to steps with the more-involved leg in the PAS condition. The IBM SPSS 23 was used for all statistical analyses. An alpha level of 0.05 was used to test statistical significance.

## Results

### Behavioral Responses to Perturbations

Across all trials, one control participant resisted the pull and did not step on the first trial, otherwise all participants stepped in all trials. Two participants (one individual post-stroke and one control) needed external assistance to regain their balance during one trial and those two trials were dropped from the analyses.

**Figure 3:**
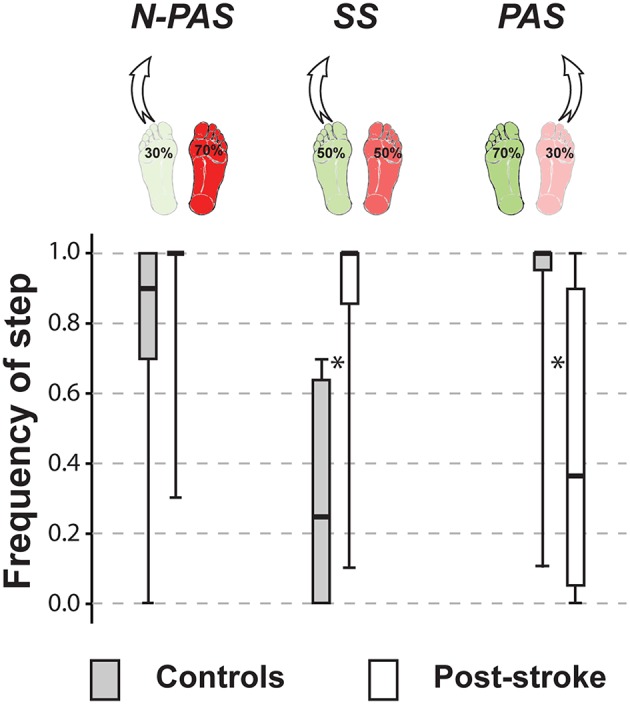
Frequency of step. The median (thick line) of the step frequency is presented for each group and each condition in which they were compared. Boxes represent 25 and 75th percentile. Bars represent min and max values. *Indicates a difference between groups at *p* < 0.05. (SS: U = 16.5, Z = −4.568, *p* < 0.001; PAS: U = 46.5, Z = 3.613, *p* < 0.001).

The control group stepped more often with their non-preferred stance leg (68.5%) in the SS condition (5 persons used that leg only); whereas in the asymmetrical conditions, they stepped more often with the leg initially supporting less weight, which was the preferred leg in the N-PAS condition (79.1% - 6 persons used that leg only) and the non-preferred leg in the PAS condition (92.4% - 12 participants used that leg only) (Figure 3). The post-stroke participants stepped with their more-involved/non-preferred leg only 11.5% of the trials in the SS condition (13 participants never used that leg) and only 6.1% in the N-PAS condition (16 participants never used that leg). However, in the PAS condition (i.e., when standing with more weight on the less-involved / preferred stance leg) they increased the use of the opposite more-involved leg to step (46.2%) and only 5 patients never stepped with that leg (Figure 3).

A significant difference was observed between groups for the number of steps taken when stepping with the preferred leg in both the SS (U = 48, Z = −2.560, *p* = 0.009) and N-PAS condition (U = 60, Z = −3.0, *p* = 0.002). Post-stroke participants took more steps (SS: Mdn = 2.40 [2.11 3]; N-PAS: Mdn = 2.53 [2 2.9]) than controls (SS: Mdn = 2 [2 2.33]; N-PAS: Mdn = 2 [1.89 2]) to regain balance. They also took significantly (U = 31, Z = −3.518, *p* < 0.001) more steps (Mdn = 2.78 [2 3]) than controls (Mdn = 2.00 [1.27 2]) when stepping with the non-preferred/more-involved leg in the PAS conditions.

### Difference Between Groups

#### APA Characteristics

Postural activity before stepping (i.e., APA) was observed in most trials for both groups of participants (96% for controls and 94% for post-stroke participants). APA was absent in 54 trials: 17 trials (3.5%) involving 7 controls and 37 trials (6.2%) involving 15 post-stroke participants.

**Figure 4:**
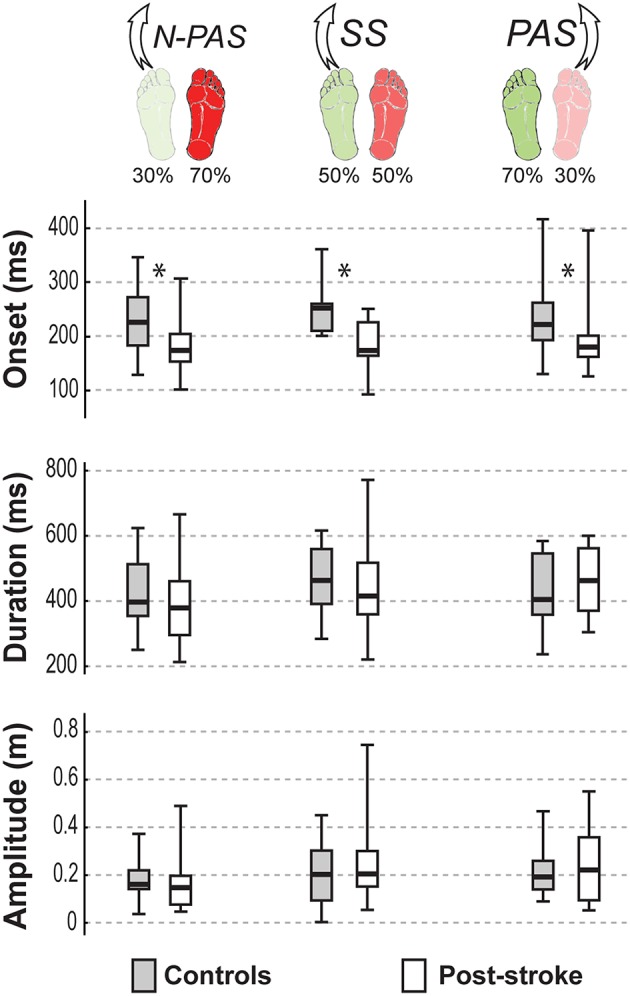
APA characteristics by group. The median (thick line) of each anticipatory postural adjustment's characteristics is presented for each group and each condition in which they were compared. Boxes represent 25 and 75th percentile. Bars represent min and max values. *Indicates a difference between groups at *p* < 0.05.

Figure 4 shows the characteristics of the APA for both groups of participants in the analyzed conditions. When stepping with the preferred stance leg, there was no significant difference between groups for APA duration (U = 86, Z = 0.616, *p* = 0.538) or amplitude (U = 98, Z = −0.088, *p* = 0.930) in SS condition or in N-PAS condition (duration: U = 112, Z = 1.267, *p* = 0.205; amplitude: U = 121, Z = 0.967, *p* = 0.334). This indicates that, when post-stroke participants stepped with their less-involved leg, the step preparation had the same characteristics as controls. However, APA onset occurred significantly earlier in post-stroke participants compared to controls in both the SS conditions (U = 26, Z = 3.256, *p* = 0.001) and N-PAS (U = 76, Z = 2.467, *p* = 0.014).

When stepping with the non-preferred leg in the PAS condition, there was also no significant difference in APA duration (U = 97, Z = −0.909, *p* = 0.363) or amplitude (U = 116, Z = −0.158, *p* = 0.874). However, APA onset occurred earlier post-stroke compared to controls (U = 63, Z = 2.253, *p* = 0.024).

#### First Step Characteristics

**Figure 5:**
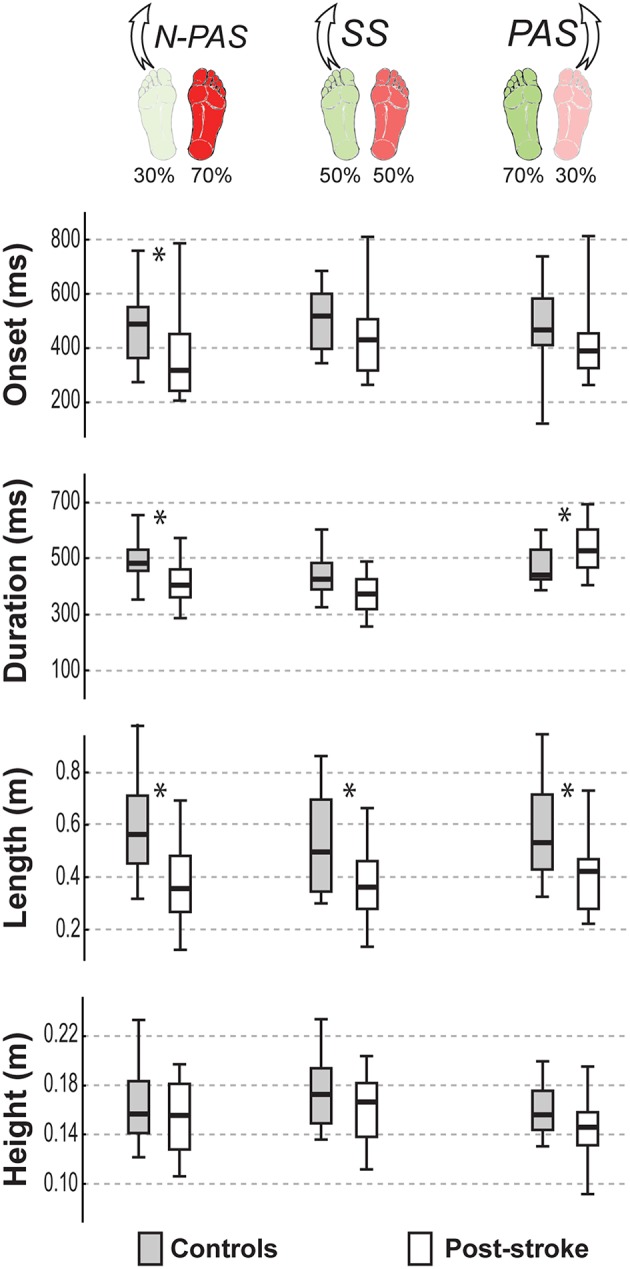
Step characteristics by group. The median (thick line) of the step characteristics is presented for each group and each condition in which they were compared. Boxes represent 25 and 75th percentile. Bars represent min and max values. *Indicates a difference between groups at *p* < 0.05.

Figure 5 shows the main characteristics of the first step for both groups of participants in the analyzed conditions. When stepping with the preferred stance leg, step characteristics were not significantly different between groups in the SS condition for most of the step variables (onset: U = 73, Z = 1.528, *p* = 0.127; duration: U = 61, Z = 1.716, *p* = 0.086; height: U = 87, Z = 0.950, *p* = 0.342). However, the step length (U = 59, Z = −2.241, *p* = 0.025) and step width (U = 59, Z = 2.11, *p* = 0.035) were different between groups showing that post-stroke participants took shorter and slightly more lateral (Mn = 1.52 cm [−0.05 2.96]) steps than controls (Mn = −0.05 cm [−1.51 1.25]). In the N-PAS condition, step onset was earlier (U = 77, Z = 2.433, *p* = 0.015), step length (U = 52, Z = −3.267, *p* = 0.001) and step duration (U = 79, Z = 2.367, *p* = 0.018) were shorter for the post-stroke participants compared to controls. No significant difference was found for step height (U = 124, Z = 0.950, *p* = 0.342) or width (U = 124, Z = 0.867, *p* = 0.386).

When stepping with the non-preferred leg in the PAS condition, step duration was significantly longer (U = 60, Z = −2.372, *p* = 0.018) and step length was significantly shorter (U = 59, Z = −2.411, *p* = 0.016) for post-stroke participants compared to controls. No differences were found for other step characteristics in this condition (onset: U = 71, Z = 1.937, *p* = 0.053; height: U = 78, Z = 1.660, *p* = 0.097; or width: U = 91, Z = 1.146, *p* = 0.252).

#### Landing Characteristics

**Figure 6:**
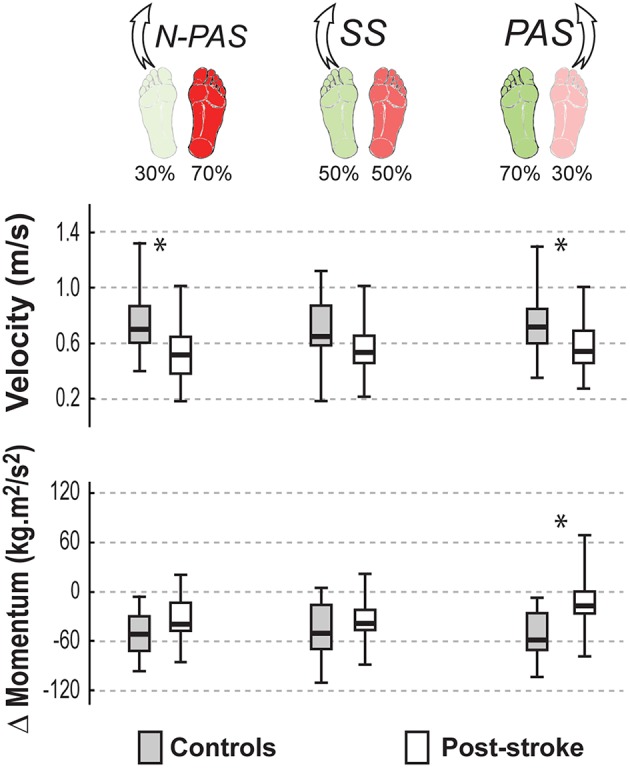
Landing characteristics by group. The median (thick line) of the landing characteristics is presented for each group and each condition in which they were compared. Boxes represent 25 and 75th percentile. Bars represent min and max values. *Indicates a difference between groups at *p* < 0.05.

Figure 6 shows the main characteristics of the landing for both groups of participants in the analyzed conditions. When stepping with the preferred stance leg in the SS condition, there was no difference between groups either in the body's velocity at first heel strike (U = 68, Z = 1.57, *p* = 0.116) or in the change in momentum between the first and the second step (U = 73, Z = −1.356, *p* = 0.175).

Asymmetrical conditions uncovered differences between groups. When stepping with the preferred leg in the N-PAS condition, the velocity at the end of the step was smaller for post-stroke participants compared to controls (U = 66, Z = 2.65, *p* = 0.008) and the change of momentum between first and second steps (U = 92, Z = −1.752, *p* = 0.08) tended to be smaller for post-stroke participants.

When stepping with the non-preferred leg in PAS condition, the velocity at the end of the step was smaller (U = 45, Z = 2.122, *p* = 0.034) and there was a smaller change of momentum (U = 34, Z = −2.665, *p* = 0.008) between first and second steps for post-stroke participants compared to controls indicating that post-stroke participants were less efficient arresting the body momentum.

### Difference Between Legs

No significant difference between legs was found for controls in postural activity preceding the step (*p* > 0.38), for stepping measures (*p* > 0.53) or the landing characteristics (*p* > 0.15). The only almost significant difference concerned the step width (Z = 1.87, *p* = 0.062). Controls tended to place their foot slightly more medially when stepping with the preferred leg (Mdn = 1.51 cm [−0.6 2.96]) than when stepping with the non-preferred leg (Mdn = −0.20 cm [−1.29 2.03]).

**Figure 7:**
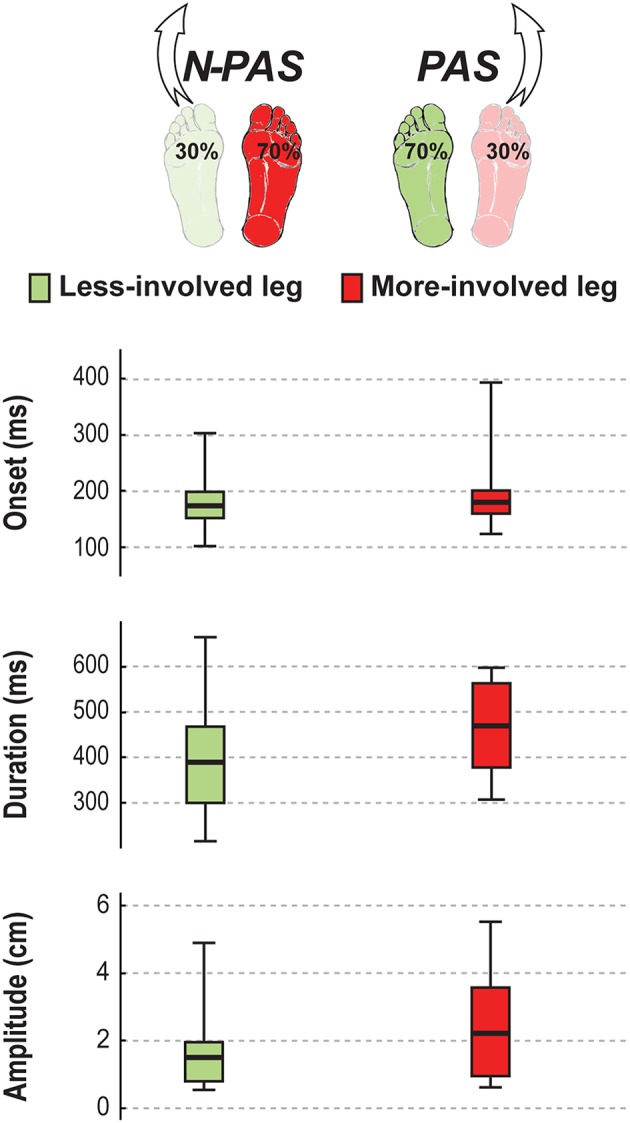
APA characteristics in stroke. The median (thick line) of the anticipatory postural adjustment's characteristics is presented for the stroke participants when stepping with the less-involved leg in the non-preferred stance condition (N-PAS) and with the more-involved leg in the preferred stance condition (PAS). Boxes represent 25 and 75th percentile. Bars represent min and max values.

**Figure 8:**
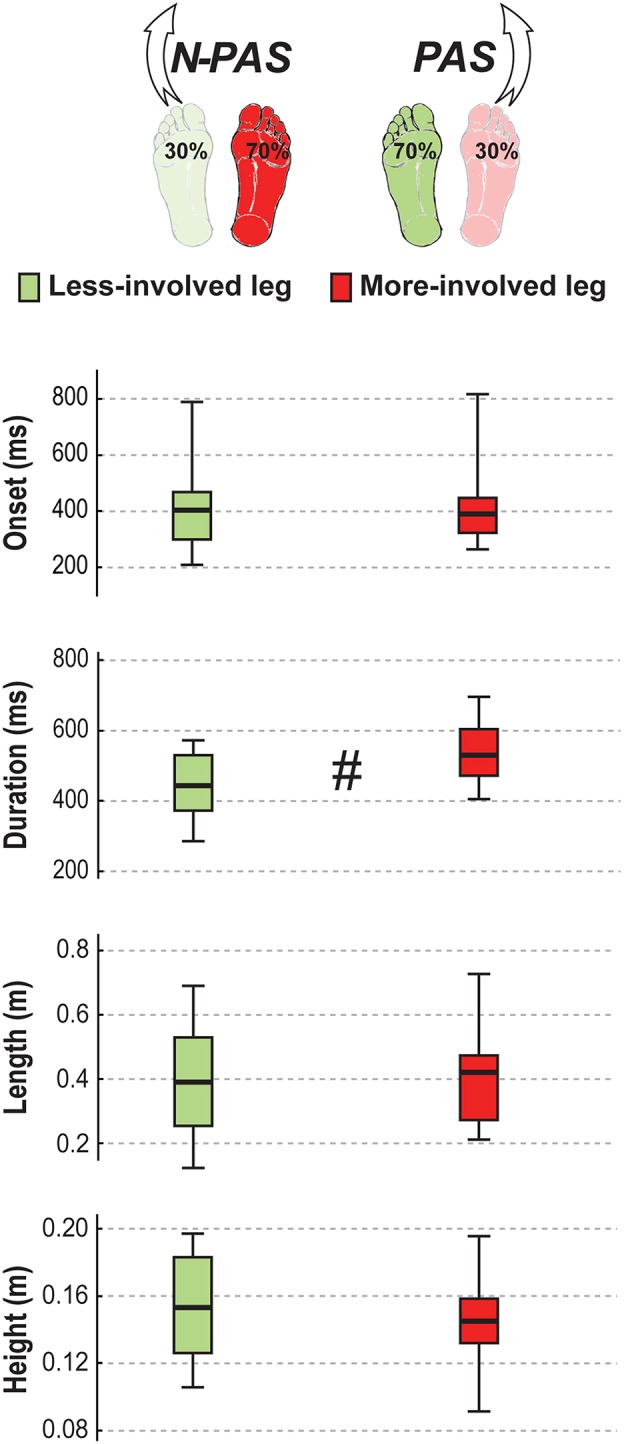
Step characteristics in stroke. The median (thick line) of the step characteristics is presented for the stroke participants when stepping with the less-involved leg in the non-preferred stance condition (N-PAS) and with the more-involved leg in the preferred stance condition (PAS). Boxes represent 25 and 75th percentile. Bars represent min and max values. ^#^ Indicates a difference between leg at *p* < 0.05.

**Figure 9:**
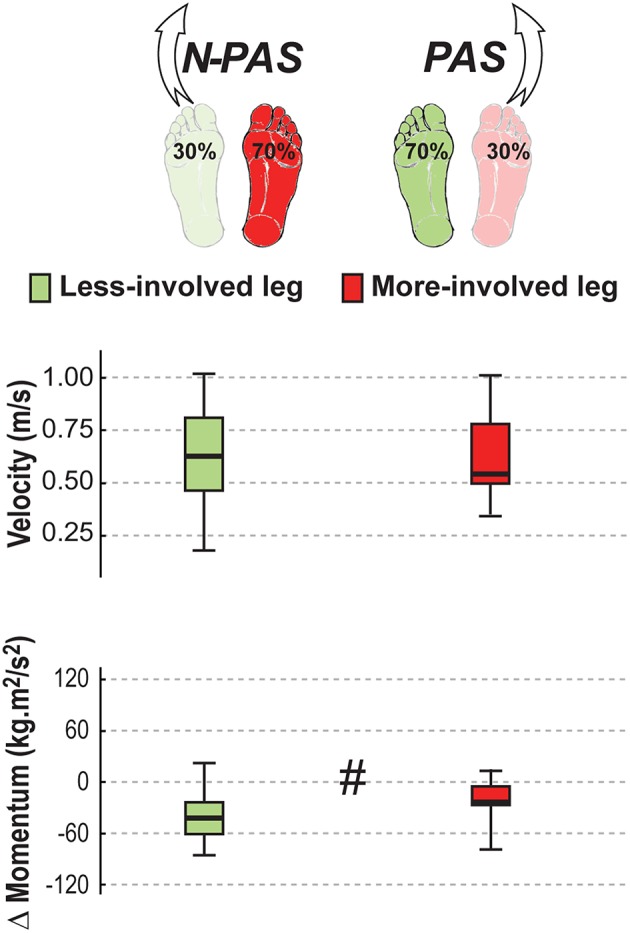
Landing characteristics in stroke. The median (thick line) of the landing variables is presented for the stroke participants when stepping with the less-involved leg in the non-preferred stance condition (N-PAS) and with the more-involved leg in the preferred stance condition (PAS). Boxes represent 25 and 75th percentile. Bars represent min and max values. ^#^ Indicates a difference between leg at *p* < 0.05.

For post-stroke participants, the APA onset (Z = 0.45; *p* = 0.65) and amplitude (Z = 1.19; *p* = 0.23) before the step were the same between the legs but APA duration tended to be longer (Z = 1.76, *p* = 0.08) when stepping with the more-involved leg compared with the less-involved leg (Figure 7). No significant difference was found between legs for step onset (Z = 0.91, *p* = 0.363), step width (Z = 1.59, *p* = 0.112), step height (Z = 1.13, *p* = 0.256) or step length (Z = 0.51, *p* = 0.609). However, a significant difference was found in number of steps (Z = 2.45, *p* = 0.014) and for step duration (Z = 3.07, *p* = 0.002). Post-stroke participants took more steps (Mdn = 2.78 [2 3]) when stepping with the more-involved leg than when stepping with the less-involved leg (Mdn = 2.3 [2 2.8]) and step duration was longer with the more-involved leg compared to the less-involved one (Figure 8). Finally, although the whole-body velocity at first heel strike was the same (Z = 0.863, *p* = 0.388), the change in momentum was significantly smaller (Z = 2.589, *p* = 0.010) when stepping with the more-involved leg compared to the less-involved side in participants post-stroke. Thus, although the body was moving at the same speed at the end of the first step, post-stroke participants had difficulties in reducing the body momentum (Figure 9).

## Discussion

This pilot study was possibly the first to examine the potential differences during induced reactive stepping in the anterior direction between post-stroke and control participants, and between legs in the post-stroke participants. As hypothesized, differences were found between post-stroke participants and controls for the non-preferred leg: steps were longer in duration and the change in body momentum following the first step was smaller despite a smaller velocity of the body at heel strike thus requiring additional steps. However, differences were also found between groups for the preferred leg: APA onset and step onset were earlier and step length was shorter. Difference in selection of the stepping leg was also seen in asymmetrical conditions allowing for a better comparison between legs in post-stroke participants. Steps with the more-involved leg were longer in duration compared to the less-involved leg and also displayed a smaller change in body momentum, thus requiring more additional steps. The findings provide several new insights regarding the stepping strategies of persons post-stroke in response to a balance perturbation and illustrate the complexity of perturbation-induced stepping performance with either leg after a stroke.

### Stepping With the Less-Involved Leg: A Preset Strategy

Stepping with the less-involved (preferred) leg was the strategy observed most often among the post-stroke group as previously reported (18, 19), particularly during symmetrical stance or when more weight was initially placed on the more-involved leg. In these cases, post-stroke participants reacted faster to the postural perturbation as illustrated by an earlier APA and step onset. This is consistent with previous studies that also showed faster initiation timing for reflex-like induced stepping in healthy elderly individuals with past falls (12, 44). It may reflect instability to the perturbation and/or anxiety about falling that possibly heightens a fear potentiated triggering of an earlier stepping response. This would thus correspond to a predetermined strategy to step earlier at the time of the perturbation, rather than waiting for a specific evaluation of the evolving conditions of instability based on sensory information. The lower ABC score illustrating lower balance confidence found in the post-stroke group compared to the controls is in line with this hypothesis. In this group of chronic stroke survivors, the decision to step could also be indicative of a learned behavior that stepping in response to the perturbation is functionally more effective than a feet-in-place response, similar to that found in healthy older adults (45). Other studies in older adults have also shown that reactive induced steps are often taken well before the limits of stability exceed the BOS (43, 45–48).

In the asymmetrical stance condition, a shorter step was observed in the post-stroke group compared to controls when they stepped with the less-involved leg and more weight was on the more-involved leg. Standing with greater weight on the more-involved leg is often challenging for people post-stroke and can be observed clinically. The shorter step length in this condition may reflect not only a desire to return to bipedal stance as soon as possible, but possibly lateral instability on the more-involved stance side during the step. In this case, the generation of hip abduction torque is an important contributor to the regulation of mediolateral stability during the single limb stance phase of stepping as well as for ongoing gait (49). Deficits in hip abduction torque associated with hip extension after stroke would possibly contribute to the reduction of step length (50).

Although we anticipated that stepping with the less-involved leg in post-stroke participants would be similar to that of controls, it was not the case. Despite a smaller velocity of the body at the end of the first step and similar change in momentum, post-stroke participants took more than two steps to stop their forward progression when using the less-involved leg whereas the controls stopped generally with <2 steps. Thus, the need for additional steps might be related to instability arising at the end of the second step when the more-involved leg contacts the ground, creating the necessity for additional steps.

### Stepping With the More Involved Leg: Issues at All Phases

Stepping with the more-involved leg was achieved by changing the initial weight distribution and initially reducing the amount of weight on that leg. The longer step duration and shorter step length of the more-involved leg in post-stroke participants compared to controls is not surprising given the greater sensorimotor impairments of hemiparetic side. The lack of differences between groups and legs for the other step characteristics when stepping with the more involved leg was unexpected. Given that the perturbation was standardized and the velocity at landing was not significantly different between legs, the problem for stepping with the more-involved leg was likely not directly related to the execution phase of the reactive step. Instead, the smaller change in momentum between heel strikes for the post-stroke participants suggested that they were less able to slow the momentum of the body between the first step with the more-involved leg and the second step, and thus that the control of the landing phase of the first step was problematic. This could be influenced by decreased extension strength or control in the more involved leg (51, 52) that is needed at step landing. This diminished extension control is noted in the differences between groups and between legs in the Upright Motor Control test which assessed the ability to perform leg extension from a flexed standing position.

When using the more-involved leg, no group differences in the APA duration and amplitude were observed. However, the comparison was made in the asymmetrical condition when the stepping leg supported only 30% of the body weight and thus when the need for weight transfer postural requirements were minimized. Despite the lack of differences between the groups, a deficit in pre-step mediolateral postural control when stepping with the more-involved leg cannot be ruled out. Specifically, when comparing the legs in post-stroke participants for the same weight distribution condition, the pre-step postural adjustment tended to be longer for steps with the more-involved leg compared to steps with the less-involved leg. Even though the legs were partially unweighted, additional time might be needed to complete the postural adjustment before a step with the more-involved leg thus illustrating deficits in postural control. This may result from the larger variability in the amplitude of the APA. In contrast with controls or less-involved leg steps, several of the post-stroke participants displayed multiple peaks in their APA performance before reaching their maximum amplitude prior to releasing a step with the more-involved leg. This observation resembled that of previous studies showing differences in postural adjustments during gait initiation between post-stroke and control participants (53) and between the more and less involved legs (2). It is quite possible that the difference in APA duration is related to challenge of the mediolateral postural adjustment prior to the release of the step.

Post-stroke participants took more steps when stepping with the more-involved leg than with the less-involved one. The need for more than two reactive steps when stepping with the more-involved leg may be less about the step execution and more about the postural challenge before the step and/or at landing. The smaller change in momentum (see Figure 9) observed between the first step with the more-involved leg and second step tend to support this hypothesis. Thus, the use of additional steps appeared to be related to difficulties in slowing the body when the more-involved leg lands on the ground. This underscores the need to consider all phases of perturbation induced stepping in the design of intervention programs targeting the specific deficits in lower limb motor control problems that occur following stroke and the need to facilitate the interactive use of both legs for balance recovery through stepping.

### Using Asymmetrical Stance for Enhancing Performance of the More-Involved Leg

The increased use of the more-involved leg for stepping when initially bearing 30% of body weight support illustrates the difficulty of releasing a step when more weight is on the leg and may reflect an underlying issue with the postural demands prior to stepping. It is thus important to implement situations facilitating the use of the more-involved leg by varying (reducing or increasing) postural demands prior to the step. In a prior study using diagonal anterior waist-pull perturbations, a pull toward the less-involved leg appeared to force increased stepping with the more-involved leg (20). This diagonal pull assisted with the weight shift off of the more-involved leg prior to stepping and facilitated increased use of the limb.

As a therapeutic approach, induced step training has been used in other populations and has shown improvement in step initiation timing in healthy older and younger adults for both reactive and voluntary stepping (54), and increased in step length in Parkinson's (55). After stroke, induced step training has been found to decrease the step completion time in one acute stroke survivor (56), and increase use of the more-involved in chronic post-stroke participants (57). Based on these results, it is conceivable that perturbation-induced step training impacts the simultaneous roles (support and stepping) of both legs in the ambulatory post-stroke individuals. In this regard, one intervention strategy should focus on increasing initiation of steps with the more-involved leg which will not only challenges the postural adjustment prior to stepping but also encourage development of strategies to control the momentum of the body at landing and subsequent steps. This could be accomplished by systematically modifying the initial stance symmetry as seen in this study or through use of cueing to step with the more-involved leg as done in other studies (23, 24, 57). Another intervention strategy should focus on training the less-involved leg to take a larger step which would facilitate weight shift and a longer single limb stance phase on the more-involved leg to enhance mediolateral stability. This could be done with visual targets or cues for foot placement. These approaches would address the bipedal nature of perturbation induced stepping and could potentially enhance the effectiveness of stepping with either leg.

### Limitations of the Study

This pilot study results are limited to community dwelling ambulatory chronic post-stroke survivor's reaction to sudden anterior perturbations of balance in controlled conditions. Due to the unrestrained responses of the participants, differences between legs for all stance symmetry posture could not be analyzed. Although the laboratory setting tries to mimic real life perturbation situations, only the onset of the pull was unknown whereas the perturbation direction was predictable. Lastly, specific neuromuscular measures were not taken so we can only speculate on the precise underlying neuromotor factors that may contribute to these findings. The findings however illustrate several issues with reactive stepping that involve both legs post-stroke.

## Conclusions

These results highlight an altered stepping strategy involving both legs in persons post-stroke and illustrate the complexity of perturbation-induced stepping with either leg. Stepping with the more-involved leg can be facilitated by unweighting the leg which constitutes a simple intervention to encourage stroke survivors to use their weaker limb. The predilection for initiating a step with the less-involved leg may be, at least in part, a learned behavior as stepping with either leg after a perturbation appears to be challenging for ambulatory stroke survivors. Therefore, consideration of the simultaneous roles (support and stepping) of both legs during reactive stepping is important for reactive balance training and should be included when designing rehabilitation approaches to enhance balance function.

## Author Contributions

KM designed and collected the data, conducted the data analysis, and drafted the initial manuscript. M-LM assisted with design, data analysis, and manuscript revisions. MR, MB, and MC assisted with results interpretation and gave critical comments for the manuscript. All authors read and approved the final manuscript.

### Conflict of Interest Statement

The authors declare that the research was conducted in the absence of any commercial or financial relationships that could be construed as a potential conflict of interest.
